# Progress and challenges in modelling country-level HIV/AIDS epidemics: the UNAIDS Estimation and Projection Package 2007

**DOI:** 10.1136/sti.2008.030437

**Published:** 2008-07-22

**Authors:** T Brown, J A Salomon, L Alkema, A E Raftery, E Gouws

**Affiliations:** 1 Population and Health Studies, East-West Center, Honolulu, HI, USA; 2 Harvard University Initiative for Global Health, Cambridge, Maryland, USA; 3 Center for Statistics and the Social Sciences, University of Washington, Seattle, Washington, USA; 4 UNAIDS, Geneva, Switzerland

## Abstract

The UNAIDS Estimation and Projection Package (EPP) was developed to aid in country-level estimation and short-term projection of HIV/AIDS epidemics. This paper describes advances reflected in the most recent update of this tool (EPP 2007), and identifies key issues that remain to be addressed in future versions. The major change to EPP 2007 is the addition of uncertainty estimation for generalised epidemics using the technique of Bayesian melding, but many additional changes have been made to improve the user interface and efficiency of the package. This paper describes the interface for uncertainty analysis, changes to the user interface for calibration procedures and other user interface changes to improve EPP’s utility in different settings. While formal uncertainty assessment remains an unresolved challenge in low-level and concentrated epidemics, the Bayesian melding approach has been applied to provide analysts in these settings with a visual depiction of the range of models that may be consistent with their data. In fitting the model to countries with longer-running epidemics in sub-Saharan Africa, a number of limitations have been identified in the current model with respect to accommodating behaviour change and accurately replicating certain observed epidemic patterns. This paper discusses these issues along with their implications for future changes to EPP and to the underlying UNAIDS Reference Group model.

## EVOLUTION OF THE UNAIDS ESTIMATION AND PROJECTION PACKAGE

The UNAIDS Estimation and Projection Package (EPP) has been developed to assist national analysts in calculating short-term projections of HIV based on currently available evidence pertaining to their own particular epidemic situations. As such, it is designed to have minimal data input requirements and to guide the user through the estimation and projection process, making it relatively easy to use. EPP has played a central part in the routine production of global estimates. It is the tool of choice for preparing national estimates and projections in most of sub-Saharan Africa.^1^ ^2^ It has also been applied in national estimation and projection work in a number of Asian countries including Cambodia, Myanmar and Vietnam, and in some countries in the Caribbean, including Haiti and the Dominican Republic.^3^

There have been four versions of EPP released publicly by UNAIDS, with the most recent being EPP 2007, described in this paper. The package has evolved over time to meet new needs as they have become clear from in-country experience, and EPP 2007 continues this evolution. The first public version of EPP in 2001 was targeted at generalised epidemics and contained only urban and rural components. Many countries found this constraining; so EPP 2003 introduced the ability for users to define their own epidemics, in terms of locally relevant geographic regions, subpopulations or a combination thereof.^4^ This extended EPP’s flexibility to deal with both generalised and concentrated epidemics. As surveillance systems expanded, a need became apparent for techniques to adjust the fitting procedures for the continuing addition of lower prevalence sites over time. Thus, EPP 2005 introduced the concept of the level fit, which is described in detail by Brown *et al*,^5^ and provides a formal method to account for the changing mix of sites. It also added turnover in surveillance populations to allow for populations that were not closed, such as sex workers or injecting drug users; and it allowed for calibration of the final curves to a single general population survey, as availability of such surveys expanded in high prevalence countries. EPP 2007 preserves or expands on all of these features.

## AN OVERVIEW OF EPP 2007 AND ITS NEW FEATURES

**Figure 1:**
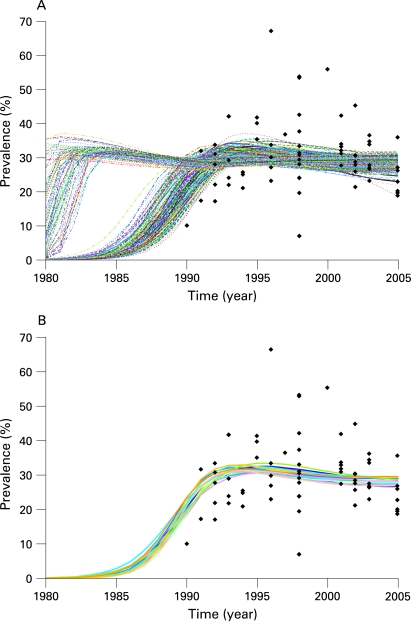
(A) The 250 best fitting curves for the surveillance data points shown in black from a sample of 25 000 randomly generated combinations of the parameters *r*, *f_0_*, *t_0_* and *φ* in the UNAIDS Reference Group model. (B) Of the original sample of 25 000 curves, the 20 shown include the best-fitting curve (defined by the maximum likelihood) and those that are statistically indistinguishable from the best fit. This example illustrates how multiple combinations of the Reference Group model parameters may fit a given data set with approximately equal validity.

EPP works by fitting a simple epidemiological model developed by the UNAIDS Reference Group on Estimates, Modelling and Projections to observed surveillance data using a maximum likelihood method. The model, described in detail elsewhere,^5^ ^6^ includes four parameters: *r*, the rate of growth of the epidemic; *f_0_*, the fraction of the population at risk of infection at the start of the epidemic; *t_0_*, the start year of the epidemic; and *φ*, a parameter that modulates recruitment to the at-risk population in response to mortality-driven declines in this population over time. One of the things that became clear from extensive experience applying this model in the field was that multiple sets of values for these four parameters could fit the data with similar likelihoods—that is, similar statistical probability. Thus, given the large inherent uncertainties in existing surveillance data, many possible parameter combinations could produce epidemic trends with approximately equally valid fits to a given set of data. For example, figure 1A shows the 250 best-fitting curves for urban sites in a southern African country, drawn from a sample of 25 000 randomly generated combinations of *r*, *f_0_*, *t_0_* and *φ*. If one identifies the curve with the maximum likelihood—that is, the best-fitting curve of the set, then another 19 curves are observed to have likelihoods that are virtually indistinguishable from that of the best-fitting curve, under the assumption that likelihood ratio statistics for two curves have an approximate χ^2^ distribution. These 20 curves are shown in figure 1B. The spread observed in these statistically similar curves gives one indication of the uncertainty in fitting the Reference Group model to observed data.

Alkema *et al*^7^ took this observation one step further, developing a methodology based on a statistical technique known as Bayesian melding for actually estimating this uncertainty. The application of Bayesian techniques to the Reference Group model is described in detail elsewhere.^7^ ^8^ In short, by generating a large number of possible combinations of the Reference Group model parameters and evaluating their statistical fit to the observed surveillance data, it is possible to give estimates of the uncertainty in the best-fit curve in the form of 95% confidence bounds. EPP 2007 has implemented this capability for generalised epidemics, and the next section describes the user interface for this component in detail. In low-level and concentrated (LLC) epidemics additional sources of uncertainty exist, largely related to the size of at-risk populations; EPP at present does not try to assess uncertainty for these epidemics. However, the same approaches *can* provide users in LLC epidemics with some idea of the range of possible curves that might be consistent with their observed data, even if it is impossible to give formal statistical bounds. Thus, an “initial guess” feature was implemented for LLC epidemics based on the same Bayesian melding elements applied formally in the generalised epidemic models.

In order to implement these approaches, which require extensive calculations and multiple simulations of the Reference Group model for each application, substantial effort was put into speeding up the EPP computational algorithms and reducing the memory requirements—both in the implementation of the model itself and in the implementation of Bayesian melding. This means that fitting techniques and other calculations in EPP 2007 are significantly faster than they were in previous versions, requiring seconds instead of minutes to fit, and tens of minutes for an uncertainty analysis as opposed to hours. At the same time, some errors were corrected in the code related to using finite time steps to implement the closed-form UNAIDS Reference Group model. These produced unrealistic population sizes and deaths under certain extreme conditions.

Finally, based on user feedback, a number of user-requested enhancements were added to EPP 2007. These include: (1) the addition of a review mode, allowing the user to open, modify and make copies of a given projection without changing the original; (2) improved calibration options including the ability to calibrate to up to three national surveys, as some countries already have two separate survey prevalence estimates available; (3) the ability to adjust prevalence in countries that have not yet conducted a national population-based survey using a correction factor informed by a comparison of survey and antenatal clinic based prevalence in those countries where national surveys have been conducted (see Gouws *et al*^9^); (4) user-defined adjustments to the sensitivity of the fitting sliders on the projection page, allowing users to make more fine-tuned adjustments; and (5) a larger interface, expanding the size of the graphs and displays.

## THE UNCERTAINTY INTERFACE—INPUTS AND OUTPUTS

The default procedure used for uncertainty assessment in generalised epidemics in EPP 2007 is:

1. Randomly generate a number of curves using values of *r*, *f_0_*, *t_0_* and *φ* within a specified range for each parameter. By default, 50 000 curves are generated to keep run times reasonable during development of preliminary projections, but the user can change this. In practice, it is recommended to use at least 200 000 curves for final national models and this is stressed in all regional estimation and projection trainings.
2. Compare the curves with the data, calculating the statistical likelihood as a “goodness of fit” measure for each curve. EPP 2007 does this automatically.
3. Resample, with replacement, 3000 curves from the original set of candidate curves, with the probability of drawing each curve proportional to its likelihood. Curves with extremely small likelihoods (that is, very bad fits to the data) are not selected at all. Curves with high likelihood (that is, the better fits) are selected often in the resample. The user can change the number of curves resampled.
4. Keep only the curves that have been selected in the resampling. Normally, the number of *unique* curves selected will be much less than 3000 because those curves that fit the data most closely are selected many times.
5. For those curves kept, calculate the mean, median and uncertainty bounds (these are the confidence intervals referred to by Alkema *et al*^8^) for each year. EPP 2007 also selects a best-fit curve, called the “UA fit” in the interface, which is the maximum a posteriori (MAP) trajectory as discussed by Alkema *et al*.^7^

**Figure 2:**
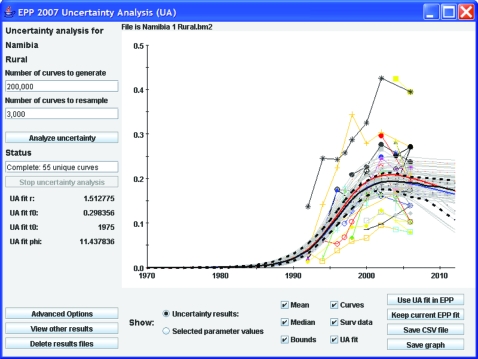
The EPP 2007 uncertainty analysis interface, showing the results for rural areas in Namibia.

Figure 2 shows the interface for implementing this procedure using rural data from Namibia. In keeping with EPP traditions, the interface has been kept relatively simple, and running uncertainty analysis only requires a user to click on two buttons if he or she is willing to accept the defaults. On the left-hand side are places for the user to enter the number of curves to generate and the number of curves to resample once the likelihoods have been calculated. Then the user clicks on “Analyse uncertainty” and EPP will run the Bayesian melding algorithm and produce the results shown in the graphical display.

Upon completion, the graphical display will contain:

1. The surveillance data, shown as separate lines with different symbols for each site. Providing individual site data give the user an indication of individual site trends and allows direct comparison of these trends against the fitted curves.
2. The uncertainty analysis fit (UA fit curve), shown as a solid red line. This is the statistically most appropriate curve (MAP trajectory) for the data as described by Alkema *et al*.^7^ The values of the four Reference Group model parameters (*r*, *f_0_*, *t_0_* and *φ*) for this best-fitting curve are displayed in the left-hand panel below the “Stop uncertainty analysis” button.
3. The yearly mean and median values computed from all the resampled curves, shown as solid blue and black lines, respectively.
4. The uncertainty bounds, shown as upper and lower broken black lines. These are calculated by determining the range in which 95% of values from the resampled curves fall for each year. These give an estimate of the uncertainty in the fit of the Reference Group model to the data.
5. The unique curves selected by the resampling, shown in light grey. This gives the user some idea of the range of curves that potentially fit the data.

The final number of unique curves that have been resampled is listed on the left side of the interface under the word “Status.” If the user is satisfied with the UA fit, then he or she clicks “Use UA fit in EPP” and EPP will transfer the result back to the projection page. It can then be used as either the final estimate or as a starting point for refining the fit further using the standard EPP fitting procedures described in earlier papers.^4^ ^5^ While the UA fit is the best parameter set of a limited sample of *r*, *f_0_*, *t_0_* and *φ* combinations, it is often possible to improve the fit iteratively by using the UA fit parameters as starting values for the EPP fitter.

**Figure 3:**
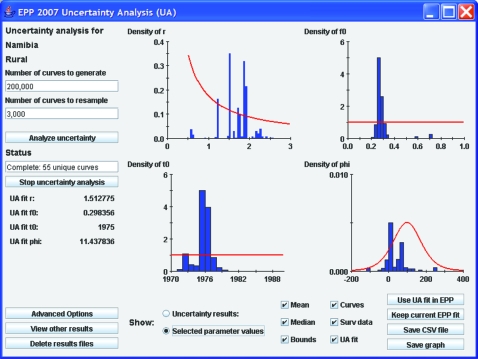
The display of the parameter values selected by EPP 2007, showing the preferred values of the Reference Group parameters in curves that were better fits to the data. The red lines represent the initial distribution sampled to generate trial values.

The interface also allows the user easy access to a graphical display of the parameters of the curves that have been selected in the resampling process. Clicking on the “Selected parameter values” button below the graphical display will bring up this display. Figure 3 shows the parameter display for the example used in figure 2. The red lines show the original distributions of the EPP parameters that were used to generate combinations for comparison with the surveillance data—that is, the prior distributions as described by Alkema *et al*.^8^ The blue histograms show the parameter values of the curves that were selected in the resampling. One can see here, for example, that there was a strong preference for values of *r* between 0.5 and 2.5, values of *f_0_* on the order of 0.3, a start year near 1976 and a *φ* value either near 0 or near 100 in the curves that were selected. If the user wishes more details on the parameters of all the unique curves selected, clicking the “Save CSV file” button will save a file, which can be read in a spreadsheet program containing detailed information about the parameters of all resampled curves and the number of times each set of parameters was resampled.

**Figure 4:**
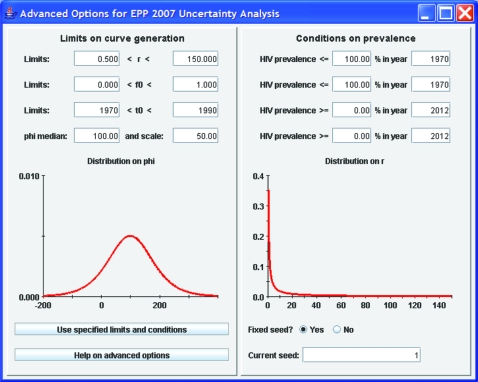
Advanced options display. This gives the user the ability to change the ranges on the UNAIDS Reference Group model parameters before generating parameter sets for trial curves. It also gives the user the ability to eliminate unrealistic curves with constraints on prevalence.

Although the functional form of the previous distributions is fixed (see Alkema *et al*^8^ for the actual prior distributions used), if the default ranges for generating parameter combinations are not appropriate for a given country, the user may change them by clicking on the “advanced options” button. This brings up the window shown in figure 4. The left-hand side of this window shows the ranges of values for the four parameters that are used in generating the initial sample of curves, and graphically displays the sampling distributions for *r* and *φ*.^8^ If the user decides some of these are inappropriate—for example, in Asia a better range of starting years might be 1980 to 2000 since epidemics there began later than in African countries, then the user can change these limits by typing in the desired values and clicking “Use specified limits and conditions”. On the right-hand side, the user can apply specific conditions on prevalence in different years. This might be of value if one finds that the random generation is selecting some curves that expert opinion says are not appropriate. An example of such problematic curves might be those on the left-hand side of figure 1A, which show possible epidemics that rise early to high levels and with unrealistic rapidity. If the epidemiological record in a country shows these are unreasonable, they could be eliminated by applying a condition such as prevalence in 1985 must be less than 10%. The “Current seed” function allows the user to vary the sets of Reference Group model parameters that are randomly generated in the uncertainty procedure. A fixed value for this seed means that the same “random” numbers will be generated on repeated runs of the same model. A fixed seed value thus enables full replicability of model results—useful for debugging country projections. However, the user is free to change the seed value manually to generate a different array of candidate parameter value sets.

For concentrated epidemics, the interface is similar except that it does not present uncertainty bounds because the techniques for estimating uncertainty in such epidemics are unclear (see final section). Instead, it presents the user with the parameters for the unique curves resampled as possible initial guesses for fitting the data provided. While EPP at present cannot estimate actual uncertainty for these epidemics, the curves presented do give the user an idea of how tightly the data constrain the possible models. The best-fitting sets of parameter values are provided in a separate window, which allows the user to enter these values on the projection page in order to explore them interactively.

**Figure 5:**
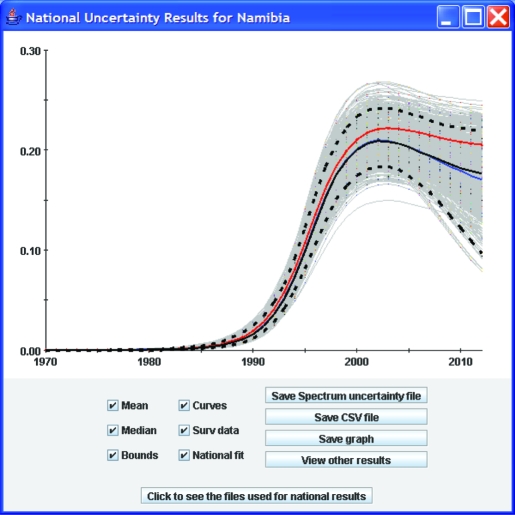
An example of combining the uncertainty from multiple projections to give an estimate of the overall uncertainty in the national projection.

EPP uses the same Bayesian melding uncertainty techniques to combine projections from different geographic areas to produce estimates of the national uncertainty. Projections from each geographical area are randomly sampled and combined 3000 times and the uncertainty bounds and a best-fit curve is generated from this resample. Figure 5 shows an example of this for Namibia. EPP generates a set of epidemic curves for both the urban as well as the rural sub-projections and combines those to generate the national epidemic curves as described by Alkema *et al*.^8^

Finally, the calibration page for generalised epidemics has added the information needed to calibrate the selected curves based on up to three national surveys. This required a shift from the overall national calibration approach used in EPP 2005 to a calibration on specific geographical subareas (in this case, urban and rural), and requires the user to input more data on the survey including standard errors and sample sizes. This calibration is described in detail by Alkema *et al*^8^ and will not be elaborated further here.

## ISSUES WITH EPP—LIMITATIONS OF THE REFERENCE GROUP MODEL

In general, in most countries EPP has worked well in fitting the surveillance data. However, as users gained experience with longer time series of data, a number of specific issues have come up in fitting EPP to some sets of country data. These include:

- Countries in which most fits to the data tend to fall to zero prevalence in the near future;
- Countries which show a steep decline followed by a levelling of prevalence;
- Countries with a long, steady decline in prevalence that is difficult to fit with the model; and
- Countries with an extended rise in prevalence that continues for many years.

One of the major limitations of the existing Reference Group model is that it assumes all parameters are constant over time. The authors have considered the potential impact of allowing changes in the parameters over time and whether this could improve the fits to the data in these more challenging countries. The two model parameters most likely to change over time are the force of infection, *r*, and the behavioural change parameter, *φ*, which determines changes in the number of people entering the at-risk population over time. Variations in *r* could account for increases in condom use, decreases in prevalence of other sexually transmitted infections acting as cofactors for HIV transmission, reductions in frequency of risky sex or similar changes. Variations in *φ* could address changes in the size of the at-risk population over time.

Variations explored included: (1) a one-time change in the value of *r* in some specified year; (2) a slower change in *r* occurring over some period requiring specification of start and end years for the change and the rate of change; (3) a change in *r* over a period coupled with a one-time change in *φ* at some point in time; and (4) allowing both *r* and *φ* to change continuously over independent time periods.

In general, allowing for more flexibility in the model made for better fits in some cases, but not in others. For example, it did not resolve the issues with the best-fit projections declining to near zero levels in certain scenarios (although allowing for a second change in *r* could address this). Most of the benefits of better fits were attainable with the models allowing for change(s) in *r*. These specifications have the advantage of being relatively parsimonious choices, only requiring the addition of two or three parameters to the model (the year of the change and the percentage of change for a one-time change in *r*, or start and end years and rate of change for continuous change in *r*) for a total of six or seven parameters. Thus, the current recommendation from the Reference Group is that this be incorporated into future versions of EPP as an advanced option, but that it will only be applied in those countries where the conventional model has extreme difficulty.

In terms of the uncertainty estimation, this will increase the computational workload substantially and may make it more difficult to make runs in a timeframe acceptable to in-country users. One possible solution to this problem is the use of incremental mixture importance sampling, proposed in a different context by Steele *et al*.^10^ In this approach, a run is made for a smaller number of curves to determine the range of values of the model parameters that are likely to fit the data. These revised ranges are then used to generate an additional more refined sample of curves to test against the data. If this is done iteratively, it will allow a larger number of curves to be generated that are closer fits to the data, while reducing the overall computational cost substantially. These approaches are being explored for possible incorporation into EPP 2009.

## EPP—FUTURE DIRECTIONS AND PROPOSED CHANGES

As outlined here, explorations continue on several ways to improve EPP and its internal model and address emerging needs. One of the problems is that the model with its fixed parameters at present is too inflexible, which may be particularly problematic in countries where major behaviour change has been occurring. The techniques discussed in the preceding section may address this to some extent, but they will probably not resolve all the issues or allow optimal fits in all countries. Patterns of behaviour change are unlikely to be characterised by monotonic increases or decreases in risk—risk behaviours may fall for a period and then begin to rise again. In the current Reference Group model, this might require multiple changes in *r*, *f_0_* and *φ* over time, but measuring those changes and incorporating them into the model in a systematic fashion would not be easy.

An alternative approach is to allow for some limited set of readily measureable behavioural inputs to influence one or two of the key parameters. For example, if the major behaviour change influencing HIV transmission is increases in condom use, changes in the force of infection parameter, *r*, made in accord with time trends in condom use might be able to address this well. This approach might also have the advantage of allowing EPP to explore alternative scenarios based on levels of programme success, which is a capability desired by countries. However, any approaches of this sort require careful examination of data availability, their robustness in fitting actual data, and their usefulness in country-level practice. This requires further study by the UNAIDS Reference Group and its collaborators.

One critical factor currently missing in EPP is the effect of antiretroviral therapy (ART) on survival and, therefore, prevalence. At present, EPP passes its national fits of prevalence to the Spectrum package for calculation of more detailed epidemiological and demographic quantities; Spectrum implements a full ART model for calculation of these effects.^11^ However, coverage of ART is getting high enough in many countries now that it will be starting to affect the observed prevalence in national surveillance systems.^12^ This means that EPP must be able to account for current ART coverage in fitting its curves to the observed data. This will also raise issues about urban/rural or population-specific differentials in ART access that need to be accounted for, an issue that requires further exploration.

An outstanding issue for future versions of EPP is the estimation of uncertainty in concentrated epidemics. Unlike generalised epidemics, where estimates are based on prevalence data from women attending antenatal clinics, calibrated by prevalence measured in general population-based surveys, in concentrated epidemics the prevalence data need to be collected from populations such as female sex workers, men who have sex with men and injecting drug users. These populations are often hidden and more difficult for surveillance systems to access in a systematic fashion.^13^ As a result, factors to consider in developing an approach for uncertainty estimation in these epidemics include large uncertainties in size estimates for these subpopulations, the representativeness of surveillance data and geographical limitations of the data, which are often available from only a small number of urban sites. The UNAIDS Reference Group on Estimates, Modelling and Projection will consider this issue in the coming year for possible incorporation into EPP 2009.

In conclusion, as it has since its inception, EPP will continue to evolve to become a more useful tool for countries to assess the state of their HIV epidemics. The interested reader may download the latest version of EPP 2007 from the UNAIDS website at http://www.unaids.org/en/KnowledgeCentre/HIVData/Epidemiology/epi_software2007.asp

## References

[1] Walker N Stanecki KA Brown T Methods and procedures for estimating HIV/AIDS and its impact: the UNAIDS/WHO estimates for the end of 2001. AIDS 2003;17:2215–25 14523279 10.1097/00002030-200310170-00010

[2] Walker N Grassly NC Garnett GP Estimating the global burden of HIV/AIDS: what do we really know about the HIV pandemic? Lancet 2004;363:2180–5 15220043 10.1016/S0140-6736(04)16511-2

[3] Ministry of Health HIV/AIDS estimates and projections 2005–2010. Hanoi, Vietnam: Ministry of Health, General Department of Preventive Medicine and HIV/AIDS Control, 2005

[4] Ghys PD Brown T Grassly NC The UNAIDS Estimation and Projection Package: a software package to estimate and project national HIV epidemics. Sex Transm Infect 2004;80 (Suppl I):i5–9 15249692 10.1136/sti.2004.010199 PMC1765839

[5] Brown T Grassly NC Garnett G Improving projections at the country level: the UNAIDS Estimation and Projection Package 2005. Sex Transm Infect 2006;82 (Suppl 3):iii34–40 16735291 10.1136/sti.2006.020230 PMC2576727

[6] UNAIDS Reference Group on Estimates Modelling and Projections Improved methods and assumptions for estimation of the HIV/AIDS epidemic and its impact: recommendations of the UNAIDS Reference Group on Estimates, Modelling and Projections. AIDS 2002;16:W1–14 12045507 10.1097/00002030-200206140-00024

[7] Alkema L Raftery AE Clark SJ Probabilistic projection of HIV prevalence using Bayesian melding. Ann Appl Stat 2007;1:229–48

[8] Alkema L Raftery A Brown T Bayesian melding for estimating uncertainty in national HIV prevalence estimates. Sex Transm Infect 2008;84 (Suppl I):i11–i16 18647860 10.1136/sti.2008.029991 PMC2569139

[9] Gouws E Mishra V Fowler TB Comparison of adult HIV prevalence from national population-based surveys and antenatal clinic surveillance in countries with generalized epidemics: implications for calibrating surveillance data. Sex Transm Infect 2008;84 (Suppl I):i17–i23 18647861 10.1136/sti.2008.030452 PMC2569190

[10] Steele R Raftery A Emond M Computing normalizing constants for finite mixture models via incremental mixture importance sampling (IMIS). J Comput Graph Stat 2006;15:712–34

[11] Stover J Walker N Grassly NC Projecting the demographic impact of AIDS and the number of people in need of treatment: updates to the Spectrum projection package. Sex Transm Infect 2006;82 (Suppl 3):iii45–50 16735293 10.1136/sti.2006.020172 PMC2576732

[12] World Health Organization, UNAIDS, UNICEF Towards universal access: scaling up priority HIV/AIDS interventions in the health sector: progress report, April 2007. Geneva: WHO, 2007

[13] Schwartlander B Ghys PD Pisani E HIV surveillance in hard-to-reach populations. AIDS 2001;15 (Suppl 3):S1–3 11421177 10.1097/00002030-200104003-00001

